# The Two Ends of My Experience with the Lymph Compound

**Published:** 1905-04

**Authors:** C. N. Palmer

**Affiliations:** Lockport, N. Y.


For Texas Medical Journal.

### The Two Ends of My Experience With the Lymph Compound.

BY C. N. PALMER M. D., LOCKPORT, N. Y.

My experience with the Roberts-Hawley Lymph-Compound covers a period of four years this present month. I had been investigating reports of cases, however, for some time previous. I had decided that my first test should be a crucial one. I will proceed to report it in brief:

Robert D———, fourteen months; suffering from malnutrition since soon after birth, from indigestion and attendant bowel difficulties, complicated during the previous few weeks with gangrene of right lung, following pleuro-pneumonia, and later the pleural effusion became purulent. The child exhibited great tenacity of life, and nutrition was fairly well sustained until the time came when suddenly the stomach rejected everything, and complete collapse followed. The child was like a wet rag. Stimulants hypodermatically seemingly had no effect except to raise temperature.

All ordinary treatment failing, the lymph was finally brought into requisition. It was administered twice daily in two-minim doses, diluted with distilled water. The first two doses were injected without any evidence of pain from the needle, and gradually the pulse became somewhat fuller, steadier and less frequent. On my arrival on the morning of the second day, the mother informed me that at 1 a. m. four ounces of food had been taken and retained, and the second bottle, about six ounces, had been consumed about an hour previous to my visit. When I entered the nursery, I found the little one sitting erect, amused with his playthings.

The treatment was continued for three months with continuous general improvement and a gradual closing up of the cavity of necrosed lung, which cavity was longitudinal, extending from the bottom of the lobe upwards several inches and giving that pronounced odor to the breath only possible in necrosed lung. A small amount of semi-purulent fluid was subsequently removed from the pleural cavity, and after due time complete convalescence ensued.

Comment upon the case seems unnecessary, except perhaps to assure the profession that this is no fairy tale, but strictly in accordance with facts developed in my first experience in the administration of the Lymph-Compound. This case is important to note, for the reason that these striking results are only possible in such extreme cases of prostration and collapse. In such cases a powerful direct cell tonic restores the balance of power, and nature reasserts herself. At the present time the little patient is in perfect health, with, of course, flattening of the affected side.

My last case, still under treatment, being a typical one, is also worthy of a permanent record. Rev. S———s, a prominent clergyman, present age 81 years. Somewhat over a year ago he was strong and vigorous, mentally and physically, and in active charge of a large parish. At this time he began to suffer with cervical suppurative tubercular lymphadenitis, one gland after another becoming involved until the posterior and right side of the neck and right submaxillary region was thickly studded with cicatrices. The case was a progressive one, new foci constantly appearing, by the involvement of new glands, health and strength gradually failing. His pastorate, to which he had been unable to give attention for several months, was at last abandoned, and about the last of May, suffering from extreme debility and prostration with great difficulty and many forebodings, he was removed to this city, and came under my charge.

His physical condition was of extreme decrepitude, emaciation and anemia; unable to walk across the room without staggering and in danger of falling, which did occur on one or two occasions. There was complete anorexia, tongue brown, dry and deeply furrowed; pulse, 120; thready; temperature, 97. There were two glandular abscesses in the neck and two in right sub-axillary region; all former abscesses cicatrized and inactive. A few days after his arrival, no doubt as a result of the journey, requiring eight or ten hours, which was very fatiguing in his critical condition, the latent cicatrices, one and all, began to discharge freely, and a new abscess appeared in the right mastoid region, so profuse, indeed, was the discharge that all dressings were soaked and clothing soiled with the effect of rapidly draining the balance of his vitality and strength. These conditions were accompanied by a temperature of 101°, much stiffness and pain in regions affected and with a decided and distressing migraine.

This was his condition when, on the 6th day of June, I began treatment with the Lymph-Compound (R.-H. Special), once, and very soon twice daily, in rapidly-increasing doses up to sixteen and eighteen minims. During the first week strychnia was administered in one-thirtieth grain doses, with gradually increasing intervals from four hours to twice daily. Since that time no adjuvant has been employed, except olive oil.

Present conditions (twenty days' treatment): Two of the cervical glands are still discharging slightly, and, upon a twenty-four-hour dressing over the abscess in the chest, there is slight discoloration. All the others have entirely subsided, also the pain and soreness. Appetite good, sleep excellent, tongue moist, slightly coated white. Digestion excellent; he eats strawberries, oranges, cucumbers, and green peas without discomfort. Pulse full, soft, compressible, 85-95 per minute; respiration normal; gaining in flesh and color; able to walk about indoors and out. Has walked three or four blocks without marked fatigue. Walks erect and firmly; has been out for a drive on several occasions. Looks good, feels good, eats good, and is in every way improving rapidly. So much for the Lymph-Compound.

This may seem to some as almost another fairy tale, but it will not seem so if they use the Lymph. I obtained, as results, in this case just what I expected, and the statement of the case is simply unvarnished, unpainted, cold, stubborn facts, and my experience with the Lymph-Compound could furnish many other cases with equally remarkable results. These marked and rapid results, however, will not obtain by the administration of the Lymph-Compound to patients of normal vigor and relatively good physical condition, although the final result with the latter class may be quite as satisfactory.

The Lymph-Compound seems to be a cell tonic, but be this as it may, it is a vitalizer and furnishes the material which nature seems to require wherewith to make repairs and also to stimulate her to the effort. At all events, whatever principal contained in the Lymph-Compound is responsible for the results obtained, or what may be the modus operandi, it has become an established fact that in well-selected cases, clinically, it does the business.

